# Supplementary material for: Synthesis of New Biscoumarin Derivatives, In Vitro Cholinesterase Inhibition, Molecular Modelling and Antiproliferative Effect in A549 Human Lung Carcinoma Cells
Source: Int J Mol Sci. 2021 Apr 7;22(8):3830. doi: 10.3390/ijms22083830 (PMC8068036; doi:10.3390/ijms22083830)

Figure S1 Effects of derivatives **12b** (a) and **12c** (b) on A549 cell metabolic activity evaluated by MTT assay. The cells were treated with the indicated concentration of the compounds for 24 and 48 h. Statistical significance * $p < 0.05$; ** $p < 0.01$; *** $p < 0.001$ for each experimental group compared to the untreated cells.

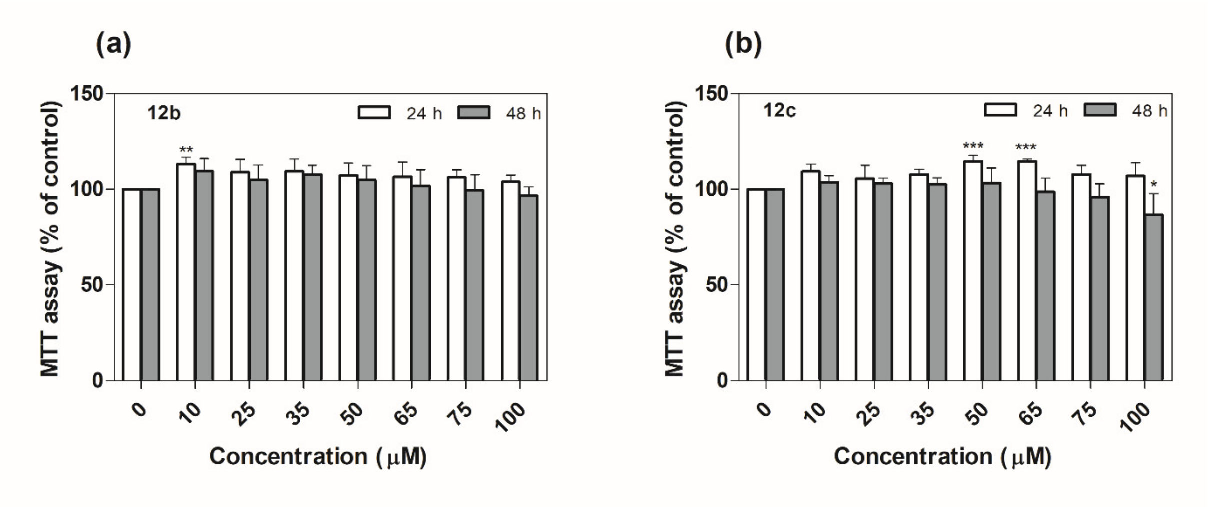

Supplement: Supplementary file 1 [file ijms-22-03830-s001.pdf]
